# A Comparative Study on the Efficiency of Two *Mycobacterium avium* subsp. *paratuberculosis* (MAP)-Derived Lipopeptides of L3P and L5P as Capture Antigens in an In-House Milk ELISA Test

**DOI:** 10.3390/vaccines9090997

**Published:** 2021-09-07

**Authors:** Sepideh Hosseiniporgham, Franck Biet, Christelle Ganneau, John P. Bannantine, Sylvie Bay, Leonardo A. Sechi

**Affiliations:** 1 Dipartimento di Scienze Biomediche, Università di Sassari, 07100 Sassari, Italy; s.hosseiniporgham@studenti.uniss.it; 2 UMR1282, Infectiologie et Santé Publique (ISP-311), INRAE Centre Val de Loire, 37380 Nouzilly, France; franck.biet@inrae.fr; 3 Unité de Chimie Des Biomolécules, Département de Biologie Structurale et Chimie, Institut Pasteur, 75015 Paris, France; christelle.ganneau@pasteur.fr (C.G.); sylvie.bay@pasteur.fr (S.B.); 4 CNRS UMR 3523, 75015 Paris, France; 5 USDA-Agricultural Research Service, National Animal Disease Center, Ames, IA 50010, USA; john.bannantine@usda.gov; 6 SC Microbiologia e Virologia, Azienda Ospedaliera Universitaria, 07100 Sassari, Italy; 7 Mediterraneam Center for Disease Control, 07100 Sassari, Italy

**Keywords:** *Mycobacterium paratuberculosis*, antibodies, ELISA, L3P, L5P, sheep, milk, bovine

## Abstract

*Mycobacterium avium* subsp. *paratuberculosis* (MAP) surface-exposed lipopeptides could be specific capture-antigen molecules targeting antibodies against MAP, in milk, through ELISA. Previous studies have revealed that MAP strains, isolated from sheep (S) or cow (C), could produce specific lipopeptides, L3P or L5P, respectively. In this study, we used L3P and L5P as capture antigens in an in-house milk ELISA (H-MELISA) to assess how these antigens perform, in comparison with other ELISA tests, on well-defined milk samples from MAP-infected sheep. The overall positivity rates of H-MELISA via L3P and L5P varied by the source of milk samples, in which, at bulk-tank-milk (BTM) level, the majority of positive cases (63.83%) reacted more against L5P, whereas a predominant number (69.14%) of milk samples were more responsive against L3P at the individual level. To clarify whether the positivity status of milk samples in H-MELISA L3P/L5P were predictive of MAP strain-types (S/C), strain-typing was carried out using PCR IS*1311*-restriction enzyme analysis. Although the presence of three MAP strains (S/C/bison types) was detected among the milk samples, the C-type (46.67%) and S-type (75%) MAP strains were detected with higher incidence among BTMs and individual milk samples, respectively. However, further examination on the H-MELISA L3P/L5P-positivity pattern of each C/S-type-MAP sample revealed that some samples had a reverse reactivity against both L3P and L5P. These results could be the consequence of either cross-reactivity between L3P and L5P (due to the similarity in the structures of the two epitopes) or simply a within-herd mixed infection with MAP strains of C and S types. These findings suggest that lipopeptide antigens could contribute a diagnostic test with optimal performance, considering the diversity of MAP strains.

## 1. Introduction

*Mycobacterium avium* subsp. *paratuberculosis* (MAP) is known as the ethologic agent of Johne’s disease (JD), an incurable gastrointestinal inflammation in ruminants [1,2] that imposes much irreparable damage on the dairy industry [3]. Animals infected with MAP often remain asymptomatic for a long period (2–6 years) [4] and can spread the disease by profuse excretion of MAP. In fact, the control and diagnosis of JD face huge difficulties due to the lack of specificity and sensitivity of diagnostic tests in the early stages of the disease. Milk production is one of the most important industries in many regions of the world, and the infection of herds with MAP may result in enormous economic losses. In addition, several studies suggested that MAP can contribute to the development of inflammatory reactions in human disorders [5,6,7] such as Crohn’s disease [8], multiple sclerosis (MS) [9], Hashimoto’s thyroiditis [10], and rheumatoid arthritis (RA) [11]. Therefore, in order to avoid any risk associated (food safety) with the presence of this pathogen in milk, improving the methods of detecting MAP in milk is essential.

To date, two primary strain types (S and C) [12] and one evolutionary intermediate (bison-type) [13,14,15,16] of MAP have been identified. The S and C types were initially defined as the indigenous strains isolated from sheep and cow, respectively, however further studies have revealed that this host orientation could not inevitably predict the strain type [16]. In fact, genotypic and phenotypic (e.g., pathogenesis and growth patterns) divergences are what discern S and C type MAP strains from each other [16,17,18,19]. The S-type MAP has further evolved into two distinct subtypes, I and III. [20]

Detection of the bacterium, regardless of the strain types, in milk and other clinical samples has been facilitated through IS*900* polymerase chain reaction (PCR) analysis. Insertion sequence IS*900* with 16–22 copies [21] in the MAP genome is one of the most sensitive and specific targets that is commonly used in PCR detection [22,23].

Insertion sequence IS*1311* is present at 7–10 copies [24] in the MAP genome. Sequence analysis revealed that multiple single nucleotide polymorphisms (SNPs) are able to discriminate the subtypes of MAP (S [I and III]/C/bison) [25]. A PCR IS*1311* followed by restriction enzyme analysis (REA) is one of the MAP strain-typing techniques that is more popular in epidemiological studies and MAP-screening programs [12,24] due to its low cost and simplicity [26].

MAP-induced antibodies in serum or milk samples could be detected by indirect enzyme-linked immunosorbent assay (ELISA), routinely used due to its rapidity, simplicity, and cost-effectiveness [27,28,29]. Up to now, some paratuberculosis ELISA tests have been introduced and evaluated [30] based on their competency in the sensitive quantifying of antibodies directed against MAP in milk samples [29]. A recent study on the efficiency of a commercial ELISA test revealed that milk ELISA could be an alternative antibody assessment-test to serum ELISA when there is no access to animal’s blood samples, since a significant level of concordance was seen between milk- and serum-ELISA at the individual-sheep level [31]. However, the agreement between serum and milk ELISA was moderate (kappa value of 0.5) in another study on dairy cows, where a relatively low-level sensitivity was found in both assays [32].

In fact, commercial milk-ELISA tests, available for detection of paratuberculosis, are mostly imperfect due to their lack of sensitivity, in particular, if the milk comes from asymptomatic animals at the early stage of JD [27,33], if there is a lack of sufficient specificity in targeting the antibodies directed against MAP, or if the rate of cross-reactivity with close mycobacterial species is not minimized [27,34]. This may underline the importance of using alternative capture-antigen molecules (as coating antigens) in ELISA that could enhance the sensitivity and specificity of the immunoassay simultaneously. Currently, two lipopeptides have been discovered in the MAP envelope that differ in C-type and subtype III of S-type [35] MAP strains, lipopentapeptide (L5P) and lipotripeptide (L3P), respectively [35,36]. The differences between L5P and L3P come from the structures of their non-ribosomal peptide synthetases (NRPSs: encoded by *mps1* gene). NRPSs take part in assembling the non-ribosomal peptide moiety of these lipopeptides, and they differ in terms of the number of modules from three to five in S and C types, respectively [35,36]. L5P has been tested successfully in the serum ELISA diagnostic test [35,36,37]. These synthetic antigens may be useful for the improvement of existing commercial tests, being specific to MAP, readily synthesized chemically as a pure product without batch-to-batch variation, and without the need for a pre-absorption step.

In a recent study, we utilized the milk qPCR and commercial milk ELISA tests to detect MAP DNA and antibodies against it in a large panel of Sardinian unpasteurized sheep milk samples, including bulk tank milk (BTM) and individual samples [31]. This sample panel represents a double opportunity to test, for the first time, the L5P and L3P lipopeptide antigens. Until now, these antigens have not been evaluated on milk samples by any study. The second advantage of this panel of samples is that it comes from animals infected with different types of MAP strains (S/C/bison). MAP strains in sheep and bovine commonly referred to as types S and C, respectively [18,19]. These S- and C-type strains of MAP have been described to express different lipopeptides on the surface of their cell walls [35,36].

In this study we compared the specificity and sensitivity of two in-house milk ELISA (H-MELISA) tests for the detection of antibodies against MAP in sheep-milk samples, using two MAP-derived lipopeptides of L3P and L5P as capture antigens. In another step, the correlation between the antibody reactivity against L3P/L5P and the type of MAP strain (S and C) was evaluated in 23 sheep-milk samples (15 BTMs and 8 individual milk samples from a MAP-infected flock [MIF] of sheep) using IS*1311* PCR-restriction enzyme analysis (REA) via *hinf*-1.

## 2. Materials and Methods

### 2.1. Bacterial Strains

*Mycobacterium avium* subsp. *paratuberculosis* strain 1515 (ATCC 43015) and *Mycobacterium smegmatis* strain MC^2^155 (ATCC 700084) were used as positive and negative controls in the study (RIVM, Bilthoven, The Netherlands). They were grown in Middlebrook 7H9 broth (Sigma-Aldrich, Milan, Italy) supplemented with 10% Oleic Albumin Dextrose Catalase (OADC; Sigma-Aldrich, Milan, Italy) and 2 mg mycobactin J (Allied Monitor, Fayette, MO, USA) and incubated at 37 °C for 3 days to 4 weeks (depending on the mycobacterial species).

### 2.2. Selection of Milk Samples

In a cohort study, a total of 128 unpasteurized sheep-milk samples, including 47 BTM samples from 41 flocks and 81 individual milk samples from a MAP-infected flock (MIF) were collected from different farms in Sardinia, Italy. These samples came from animals that were recently tested by other MAP-diagnostic assays, and corresponding data was published in detail in another work (Tables S1 and S2) [31]. Accordingly, the milk samples were distributed into two groups of negative- and MAP-positive controls, based on the positivity/negativity status of the studied animals tested by milk qPCR, commercial milk ELISA, serum ELISA, and fecal PCR. Specifically, the specimens belonging to animals that were found positive by at least one test were considered MAP-positive, whereas the healthy milk samples came from animals that showed no positivity by any of the mentioned techniques (Table 1).

### 2.3. In-House Milk ELISA (H-MELISA) on BTMs and Individual Milk Samples (MIF) Using L3P and L5P Lipopeptides

The capture antigens L3P and L5P were synthesized manually on a solid phase using Fmoc chemistry, as described before [35,36]. In brief, MAP-specific lipopeptides, L3P and L5P (Table 2), were reconstituted in ethanol 99.8% (Sigma-Aldrich, Milan, Italy), diluted in 0.05 M carbonate-bicarbonate buffer (Sigma-Aldrich; pH: 9.5) at a final concentration of 10 μg/mL, and 50 μL of which was used for coating each well of 96-well plates. Then, plates were stored at room temperature (RT) overnight, to be completely air-dried. To remove inhibitors such as lipids (cream) and somatic cells (pellet), milk samples were centrifuged at 10,000× *g* and 4 °C for 2 min, and then the whey phase (the liquid between the cream and pellet) was aspirated into a new Eppendorf tube (1.5 mL) and stored at −28 °C to be analyzed later [31].

Next, plates were coated with 100 μL phosphate-buffered saline (PBS)/0.5% (*w*/*v*) gelatin (produced from cold water fish skin; Sigma-Aldrich, Milan, Italy) and stored at 37 °C for 1 h. Later, plates were washed five times with 200 μL PBS-Tween 20 (PBST; 0.05%) and charged with 100 μL of diluted milk samples in PBS (1:2). Then, samples were homogenized using an orbital shaker (50 rpm/5 min) and incubated at 37 °C for 90 min. In addition, positive and negative controls were included in the assessment (controls came with a commercial ELISA kit of *Mycobacterium paratuberculosis* antibody test kit (IDEXX Laboratories, Westbrook, ME, USA)). Arachidic acid, as the second negative control, was also applied in order to resemble and monitor the reactivity of the lipid moieties of the lipopeptides (L3P/L5P) in response to lipid structures in milk samples. The controls were diluted in PBS-Tween (0.05%) at a final concentration of 1:20. Then, plates were washed five times with 200 μL PBS-Tween (0.05%), charged with 100 μL anti-sheep IgG H&L (HRP) (1:150000, abcam6900), and incubated at 37 °C for 1 h. This was followed by washing the plates (five times; PBS-Tween 0.05%), adding 100 μL substrate containing TMB (3,3′, 5,5′-tetramethylbenzidine; abcam 171522), and incubating the plates at RT for 13 min. Finally, the reaction was stopped by adding 100 μL of stop solution containing 5% methanesulphonic acid (abcam 171529), and optical densities (OD) were subsequently read at the wavelength of 450 nm (SpectraMAX Plus384, Molecular Devices, Sunnyvale, CA, USA).

### 2.4. Milk Culture

The viability of MAP in milk samples was assessed by a culture-based method. Briefly, 25 to 30 mL of the samples (depending on the provided volumes) was centrifuged at 5300× *g* and 4 °C for 30 min and the whey phase was discarded. Then, both the cream and pellet portions were resuspended in 25 to 30 mL of 0.75% *w*/*v* hexadecylpyridinium chloride (HPC; Sigma-Aldrich, Milan, Italy), mixed by vortex thoroughly, and incubated at RT for 4 h. This was followed by treatment with HPC, a chemical decontaminant, which can enhance the recovery of MAP from the cream fraction [38] and reduce the chance of growing microorganisms—other than mycobacteria—on the media [39]. The samples were again centrifuged at 5300× *g* and 4 °C for 30 min. After that, the supernatant was removed and pellets were resuspended in 1 mL 1× phosphate-buffered saline (PBS; pH = 7.5). Subsequently, aliquots of suspensions (200 μL) were spread on Middlebrook (MB; Sigma-Aldrich, Milan, Italy) 7H10 agar supplemented with 10% Oleic Albumin Dextrose Catalase (OADC; Sigma-Aldrich, Milan, Italy) and Mycobactin J (2 mg/L; Allied Monitor, Fayette, MO, USA) and incubated at 37 °C for 6 to 12 weeks. The plates were observed frequently and Ziehl-Neelsen (ZN) stain was used to detect acid-fast bacilli [39]. Later, DNA was extracted from ZN-positive colonies, and the sequences thereof were used as templates for qPCR IS*900* and PCR IS*1311* analyses.

### 2.5. Extraction of DNA from Milk Samples and Colonies

DNA was extracted from HPC-treated and homogenized milk samples, as described earlier, using an RTP Mycobacteria kit (Stratec kit, Stratec Molecular GmbH, Berlin, Germany) [31]. Regarding the milk samples cultured on MB 7H10 agar, the plates were observed frequently, and Ziehl-Neelsen (ZN)-positive single colonies underwent further molecular inspection, in which colonies were harvested, suspended in 1 mL 1× PBS (pH = 7.4), and centrifuged at 5300× *g* at 4 °C for 30 min. Then, the supernatant was decanted and DNA was extracted from pellets using an RTP Mycobacteria kit (Stratec kit, Stratec Molecular GmbH, Berlin, Germany), according to the manufacture’s instructions, for the extraction of DNA from sputum samples (Protocol 1).

### 2.6. Restriction Enzyme (RE) Analysis on IS1311-Targeted PCR Products and Strain Typing (S- or C-Class)

Insertion sequence IS*1311* was targeted in seven colonies and fifty-three milk samples (comprising 34 BTMs and 19 individual milk DNAs) that were IS*900*-positive and amplified by conventional PCR using primers IS*1311*-F and IS*1311*-R. The reaction mixture consisted of 0.2 μM of each primer (Table 3), 200 μM of each dATP, dCTP, dGTP, and dTTP, 1× PCR buffer, 1.5 mM MgCl_2_, 3 μL of DNA template, and PCR-grade water to a final volume of 20 μL [40]. The PCR condition was as follows: initial denaturation at 94 °C for 3 min, followed by 35 cycles of denaturation at 94 °C for 30 s, annealing at 60 °C for 30 s, and extension at 72 °C for 1 min, with a final extension at 72 °C for 10 min [41]. Eventually, the IS*1311*-positive products (608-bp bands on gel-electrophoresis [2%]) were subjected to restriction enzyme analysis using *hinf*-1. Briefly, 10–13 μL of amplicon (depending on the strength of the bands in the visualization step) was added into a master mix, including 2 μL restriction buffer 10× (RB), 0.2 μL bovine serum albumin (BSA), 0.5 μL restriction enzyme (RE), and water, to a final concentration of 20 μL. This was followed by the incubation of samples at 37 °C for 2 h and visualization by gel-electrophoresis (3%).

## 3. Statistical Analysis

Statistical analysis was carried out using R software (version 4.0.5). To estimate the best cutoff corresponding to positivity, receiver-operating-characteristic (ROC) -curve analysis and area under the curve (AUC) were carried out using various binary reference models (0,1) at the levels of the herd and individual, respectively. We recently published the data related to the reference models, which included the milk qPCR (MqPCR), commercial milk ELISA (CMELISA), serum ELISA (SELISA), and fecal PCR (FPCR), in detail, in another publication about the identification of MAP in sheep-milk samples (Tables S1 and S2) [31]. Accordingly, in the following study, the binary reference models were proposed based on the results of the MqPCR and CMELISA, in isolation and together, at the BTM level. Unfortunately, access to the animal blood and feces was impossible at the BTM level. However, at the individual level, collecting blood and feces was as feasible as collecting milk samples, by which the references were adjusted based on the results of the MqPCR, CMELISA, serum ELISA (SELISA), and the fecal PCR (FPCR), individually and together. Later, the sensitivity of selected cutoffs in the prediction of positive/negative cases was estimated based on the level of concordance that H-MELISA L3P and H-MELISA L5P had with MqPCR/CMELISA at the BTM level and MqPCR, CMELISA, SELISA, and FPCR at the individual level (MIF). In addition, Pearson correlation analysis was performed and the level of association between the H-MELISA L3P and H-MELISA L5P data sets, at the BTM and individual (MIF) levels, was computed (the statistical significance was adjusted for a *p*-value of <0.5).

## 4. Results

### 4.1. Homemade Milk ELISA with the L3P and L5P Epitopes on BTMs

Cutoffs corresponding to H-MELISA L3P- and L5P-positivity were adjusted to optical densities of 0.8895 and 0.59925, respectively (ROC-curve analysis). Accordingly, 27.66% and 63.83% of 47 BTMs were identified as being H-MELISA-positive, producing various titers of antibodies directed against the L3P and L5P epitopes, respectively. Among the negative controls determined by the results of the milk qPCR and commercial milk ELISA [31], 11.76% and 58.82% were H-MELISA-L3P- and H-MELISA-L5P-positive, respectively (Table S1). To estimate how specifically and sensitively H-MELISA L3P/L5P could act in the determination of MAP-positive and -negative controls, the efficiency of various reference models was evaluated in ROC-curve analysis. H-MELISA L3P was found to be more specific (SP: 94.74%, SN: 39.3%, AUC: 64.8%), while H-MELISA L5P was found more sensitive (SP: 41.18%, SN: 63.33%, AUC: 59.4%) under the conditions that both milk qPCR and commercial ELISA were gold standards. Subsequently, H-MELISA L3P demonstrated a significant level of concordance with both MqPCR and CMELISA, by 60%, whereas H-MELISA L5P agreed better with MqPCR (53.19%) than CMELISA (38.30%).

**Figure 1:**
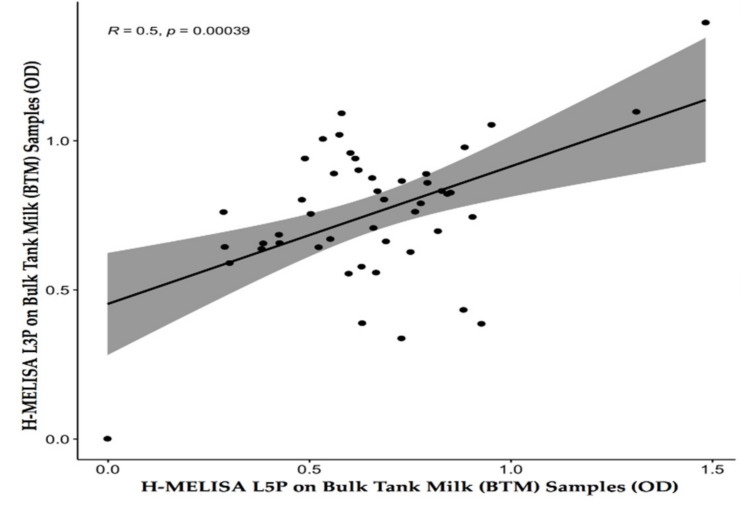
Pearson correlation test between the two H-MELISA analyses via the two MAP-derived lipopeptides of L3P and L5P on 47 bulk-tank-milk (BTM) samples (*r* [degree of freedom = 45] = 0.5, *p* = 0.00039).

Interestingly, the Pearson correlation test revealed a moderate positive association between antibody reactivity against two MAP-derived lipopeptides of L3P and L5P (*r* (45) = 0.5, *p* = 0.00039; Figure 1) in H-MELISA analysis at the BTM level.

### 4.2. Homemade ELISA with L3P and L5P Epitopes on Individual Milk Samples from a MAP-Infected Flock (MIF)

As per the ROC-curve analysis, optical densities of 0.406 and 0.513 were assigned for positivity cutoffs in H-MELISA L3P and H-MELISA L5P respectively. Among 81 individual-milk samples collected from a MAP-infected flock (MIF), 69.14% and 32.09% of cases were detected positive by H-MELISA L3P and H-MELISA L5P, respectively. Surprisingly, the majority of negative controls (71.11%; negative controls were diagnosed by all the following testes: MqPCR, CMELISA, SELISA, and FPCR (Table S2) [31]) were H-MELISA-L3P positive, whereas only 20% of negative controls were H-MELISA-L5P positive. ROC-curve analysis revealed that the type of used gold standard could affect the specificity/sensitivity of H-MELISA L3P/L5P and consequent results by area under the curve (AUC), in which H-MELISA L3P was more specific (SP: 71.93%, SN: 37.5%%, AUC: 64.2%) when both CMELISA and FPCR were binary reference models, whereas H-MELISA L5P had higher specificity (SP: 80.43%, SN: 48.6%, AUC: 59%) when MqPCR and SELISA, together, formed a gold standard. However, both H-MELISA L3P (SP: 70.9%, SN: 34.61%, AUC: 61.6%) and H-MELISA L5P (SP: 76.36%, SN: 50%, AUC: 54.5%) introduced a specificity above 70% under the condition that serum ELISA (SELISA) was a reference model. Additionally, the functionality of H-MELISA L3P/L5P in detection of MAP-positive/negative controls were estimated computing the agreement between H-MELISA L3P/L5P and the other MAP diagnostic assays, in which H-MELISA L3P almost had a moderate-to-weak correlation with SELISA (40.74%), FPCR (38.27%), MqPCR (37.04%), and CMELISA (37.04%), whereas this association was more robust between H-MELISA L5P and each of the following tests: SELISA (67.9%), CMELISA (66.67%), MqPCR (64.2%), and FPCR (60.49%) (Table S2).

**Figure 2:**
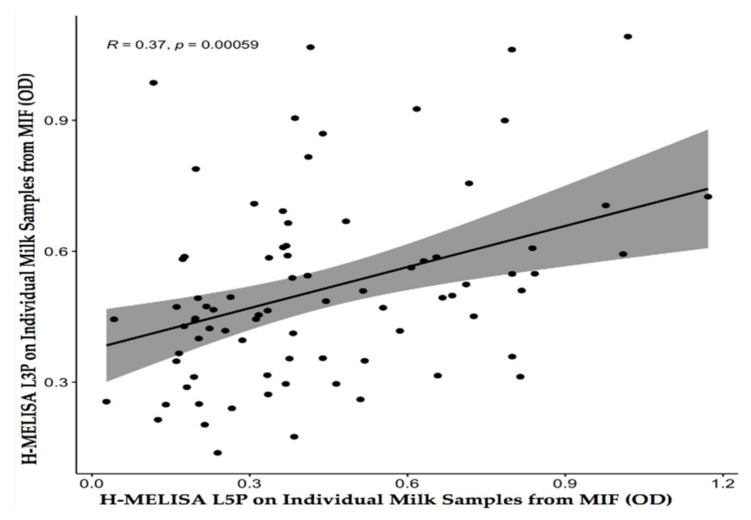
Pearson correlation test between the two H-MELISA analyses via the two MAP-derived lipopeptides of L3P and L5P on 81 individual milk samples from a MAP-infected flock (MIF) of sheep (*r* (degree of freedom = 79) = 0.37, *p* = 0.00059).

Data generated by the two homemade milk ELISA (L3P/L5P) were further analyzed by Pearson correlation test. This analysis indicated that a low positive association existed between antibody reactivity against the two MAP-derived lipopeptides of L3P and L5P (*r* (79) = 0.37, *p* = 0.00059; Figure 2) at MIF level.

### 4.3. Relationship between the Type of MAP Strain (S or C) and Reactivity against L3P/L5P in the In-House Milk ELISA (H-MELISA)

To understand whether or not the positivity status of samples in H-MELISA L3P/L5P could predict the type of MAP strains (S/C) in BTMs or individual (MIF) level milk samples, strain typing was performed on qPCR IS*900*-positive colonies and milk samples using PCR *IS1311*-restriction enzyme (RE) analysis (*hinf*-1). In fact, the amplification of IS*900* target was important prior strain typing giving us an overview about the presence/lack of MAP in samples, regardless of the type of MAP strains. However, insertion sequence IS*1311* was selected in strain typing since this region has polymorphism within subspecies and this characteristic could be exploited in restriction analysis to discriminate various types of MAP strains (S/C/bison) from each other.

**Figure 3:**
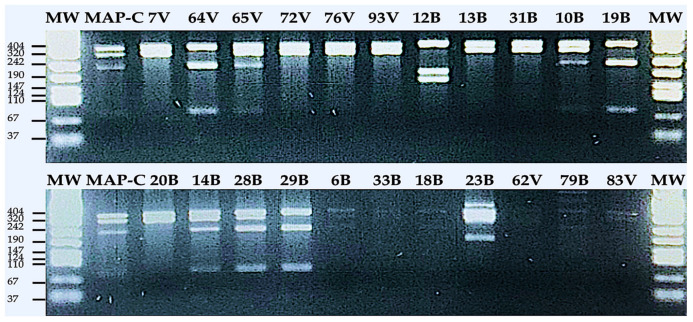
Restriction enzyme analysis (REA) on IS*1311*-positive PCR products obtained from sheep-milk samples. The first and last lanes are 100-bp marker (MW); second lane is MAP standard strain 1515 (MAP-C). The remaining lanes belong to IS*1311*-targeted amplicons from sheep-milk samples that underwent REA analysis. Samples “13B, 20B, 31B, and 33B” at the BTM level and “7V, 62V, 72V, 76V, 83V, and 93V” at the individual level (MIF) are S-type while samples “6B, 10B, 14B, 23B, 28B, and 79B” at the BTM level and “65V” at MIF level are C-type, bison type samples “18B, 19B, and 29B” at the BTM level and “64V” at MIF level were also observed. Sample 12B yielded an unusual pattern not seen before (B and V letters by the numbers are indicating BTM and individual milk sample respectively).

Seven out of 128 milk samples contained ZN-positive colonies that were qPCR IS*900*-positive. Among them, only one sample (82B) was detected positive by PCR IS*1311* as well. Restriction enzyme analysis (REA) on the PCR product demonstrated that this colony (sample 82B) is a C-type MAP, as its band pattern matched the C-type MAP used as the reference strain. Furthermore, IS*1311* was PCR amplified in 44.12% and 42.11% of IS*900*-positive BTMs and individual (MIF) milk samples respectively (23 out of 53 samples). Interestingly, IS*1311* strain typing analysis showed that all three classes of MAP (S, C, and bison) existed among Sardinian sheep-milk samples (Figure 3). The S and C types produced two similar bands at 323- and 285-bp, however C type patterns could be distinguished by two additional IS*1311* fragments at 218-bp and 67-bp in size (Table 4). In total, there were four S type strains identified in BTMs by this method. An additional six S type strains identified in individual sheep samples as well. Finally, there were eight C type strains in BTM and individual sheep (Figure 3, Table 4). Bison type was differentiated from the other strains indicating three bands at the lengths of 323-bp, 218-bp, and 67-bp [25,41] (Figure 3, Table 4). Consequently, bison type contributed the lowest quantities in both groups of BTM (18B, 19B, and 29B) and individual (MIF; 64V) with 20% and 12.5% respectively (Figure 3, Table 4).

The results of strain typing on 23 (IS*900*/IS*1311*)-positive DNAs, extracted from colonies or milk samples, revealed that C (46.67%) and S (75%) classes were the predominant strain types at the level of BTM and individual sample (MIF) respectively. Although, the overall rates of H-MELISA-L3P/L5P positivity at both the herd and individual levels were in accordance with this strain-type orientation, there were some C- and S-type samples that did not follow the expected patterns in reactivity against L3P and L5P, in which, among the four BTMs recognized as being S-type, sample 33B showed higher antibody reactivity against L3P, whereas samples 20B and 31B had similar optical densities against both L3P and L5P, and sample 13B was more responsive against L5P (Table 5). The difference between antibody reactivity of each S-type sample against L3P and L5P ranged between 0.014 ≥ OD ≥ 0.54 in H-MELISA analysis. In addition, in the same group, a weak agreement was found between antibody reactivity against L5P and identification as C-type MAP strain. In brief, two out of seven BTMs that were characterized as being C-type reacted more against L5P (samples 14B and 28B; Table 5), samples 10B and 23B had similar reaction against L3P and L5P, whereas samples 6B, 79B, and 82B were more reactive against L3P (Table 5). The difference in antibody reactivity of each C-type BTM against L3P and L5P ranged between 0.0425 ≥ OD ≥ 0.5125 through H-MELISA analysis. In contrast, a never-before-defined correlation between antibody reactivity against L3P and identification as bison-type was discovered, in which samples 18B, 19B, and 29B (Table 5), recognized as bison-type, reacted more against L3P than L5P, and the difference between antibody reactivity against L3P and L5P of each bison-type sample ranged between 0.049 ≥ OD ≥ 0.3295.

The uncertainty in the stratification of MAP strains (C or S types) based on H-MELISA-L3P/L5P positivity was also noticed in the MIF category. Three of six S-type isolates were highly reactive against L3P (samples 7V, 72V, 83V; Table 5). Samples 62V and 93V had the same titers of antibodies directed against both L3P and L5P, whereas sample 76V had a slight rise in titers of antibody against L5P rather than L3P. Interestingly, the only bison-type (64V) and C-type (65V) isolates of this category reacted more against L3P. The difference between the antibody reactivity of each of S-type, C-type, bison-type samples against L3P and L5P were estimated to be “0.0065 ≥ OD ≥ 0.3115”, “OD = 0.3085”, and “OD = 0.1325”, respectively.

## 5. Discussion

The level of antibodies directed against MAP could comparably be measured in milk samples as well as sera [42] through ELISA analysis if MAP-specific epitopes are selected [43] to capture the right antibodies and appropriate gold-standard models are employed [31] to estimate the best cutoffs for determining positivity. Previous studies declared that MAP L5P specifically induces humoral responses in MAP-infected animals [36] and this characteristic could be exploited in the immunological detection of MAP and screening animals with JD. The efficiency of MAP lipopeptides in serological detection of MAP has previously been evaluated in other studies [27,36,37]. As in a survey on the accuracy of three different ELISAs in screening paratuberculosis in sera of healthy, MAP- and non-MAP (other mycobacteria)-infected cows, L5P was employed as a capture antigen for developing an in-house ELISA assay and it detected antibodies directed against MAP in the sera of the studied animals with a significant specificity (98.9%), but less sensitivity (37%) [27]. Further sequence analysis along with biochemical and physicochemical investigations of MAP lipopeptides clarified that the structures of lipopeptides varies in MAP strains that are indigenous in sheep (S-type) and cow (C-type), in which the *mps1* gene encodes non-ribosomal peptide synthetases (NRPSs) that contribute to the production of lipopeptides in various strains (S and C types) and are constituted by five and three modules in C-type and S-type MAP strains, respectively [35]. This suggested that L5P and L3P might be ideal MAP-specific targets that could differentiate not only MAP from other mycobacteria, but also MAP strains of C-type and S-type from each other [35].

The present study, for the first time, used the MAP surface-exposed lipopeptide of L3P as a capture antigen in an in-house milk ELISA in order to screen antibodies directed against MAP in sheep-milk samples. Furthermore, the functionality of L3P was compared with the other lipopeptides (L5P) performing H-MELISA L5P on the same samples. To select the best cutoffs corresponding positivity, ROC-curve analysis was performed. Cutoffs representing positivity were selected carefully, since any miscalculation in this step could influence the specificity and sensitivity of the assay significantly [44]. The importance of using various gold standards in this study was in assessing how a reference model, both in isolation and in association with other models, could bias the cutoffs for positivity, conditional on many studies’ consensus regarding the efficiency of culture (SN: 30% and SP: 100%) [45], whereas no perfect gold standard has been created that could match all requirements of all assays in various studies [46]. The milk samples that were used in this study were previously tested by milk qPCR and commercial milk ELISA in comparison with other MAP diagnostic techniques, such as serum ELISA and fecal PCR, in our recent work [31]. Accordingly, milk qPCR, commercial milk ELISA, serum ELISA, and fecal PCR were assigned as references in determining the positivity and negativity status of each milk sample. Therefore, milk samples were distributed into two categories of MAP-positive and -negative controls. We noticed that the type of gold standard could influence the sensitivity and specificity of H-MELISA L3P/L5P at both the levels of BTM and individual samples (MIF). Specifically, H-MELISA L3P and H-MELISA L5P represented more specificity when, respectively, milk qPCR and commercial milk ELISA were the gold standard at the BTM level; whereas, at the MIF level, the most optimum specificities (above 70%) were induced to both H-MELISA L3P and L5P when serum ELISA was the binary reference model. The recent result agreed with our previous study on 128 individual milk samples from a MAP-infected sheep flock that indicated that serum ELISA (SP: 0.94, SN: 0.75; *p* < 0.0001) induced a significant specificity and sensitivity to milk-ELISA assessments [31]. Accordingly, optical densities that conferred a specificity (SP) of equal or between 70–80% and a sensitivity (SN) of above 30%, for a majority of reference models, were selected as cutoffs for positivity. These specificities and sensitivities were in the same range as those (SP: 0.83–1.00 and SN: 0.29–0.61) defined by a review study on ante mortem diagnosis of paratuberculosis in MAP-infected animals [44]. To the best of our knowledge, several factors could influence the specificity and sensitivity of H-MELISA L3P/L5P, including the sample size, the type of gold standard selected in ROC-curve analysis, and the status of Johne’s disease in animals studied here.

Our analysis depicted that the overall rates of H-MELISA-L3P/L5P positivity varied by the source of milk samples (BTM or individual). More samples were reactive against L5P and L3P at the BTM and individual (MIF) levels, respectively. Surprisingly, the majority of negative controls (their negativity confirmed by all following assays: milk qPCR, commercial milk ELISA, serum ELISA, fecal PCR) represented titers of antibodies directed against L5P and L3P epitopes in BTMs and individual milk samples, respectively. An issue that might be discussed, here, concerns why these negative controls should contain so much antibody against L3P and L5P epitopes to be characterized as being MAP-infected, while no evidence of MAP DNA was recognized in these samples. According to our analysis, MAP DNA could not be detected in a sample unless presenting at detectable concentrations. Although target IS*900* has 16–22 copies [21] in whole MAP genome that seem sufficient for molecular diagnosis, these copies could not entirely be transferred into the qPCR reaction vessels. Specifically, we reduced the volume of milk per sample required for DNA extraction to only 5 mL, in order to overcome problems raised by resource limitation and this could further reduce the presence of MAP DNA in milk samples. On the other hand, the absence of MAP DNA in milk samples could not preclude the presence of antibodies directed against MAP lipopeptides (L3P or L5P) circulating in milk and other body fluids of the corresponding animals, as MAP-infected animals are characterized by the immune responses they show at different stages of JD, such that any might be just a MAP shedder, just have humoral reactivity against MAP, or have both responses simultaneously.

In addition, the comparison between the results of H-MELISA L3P/L5P and other antibody-assessment methods depicted that H-MELISA L5P, at the BTM level, and H-MELISA L3P, at the individual level (MIF), had the lowest level of agreement with commercial milk ELISA and serum ELISA/commercial milk ELISA, respectively. This may demonstrate that H-MELISA L3P/L5P could function even better than commercial ELISA tests (milk or serum) and even detect minor titers of antibodies against MAP in milk samples. In fact, the types of capture-antigen molecules used in the structure of H-MELISA L3P/L5P and commercial ELISA tests could influence the functionality of the techniques significantly. Most commercial ELISA tests are designed to detect antibodies against crude extracts or specific epitopes of C-type MAP (as capture antigens) in milk or serum samples. Based on our interpretations in MAP-relevant infections, a noticeable proportion of antibodies against MAP are induced against its lipopeptides, since lipopeptides, as well as lipids, exist with higher frequency in the whole MAP cell structure compared with other epitopes. Therefore, we selected L3P and L5P as capture-antigen molecules in H-MELISA tests to target as many existing antibodies as possible against these epitopes in our milk samples.

Based on the positivity patterns in H-MELISA L3P/L5P, it could be interpreted that C-type and S-type MAP are dominant strains at the BTM and individual (MIF) levels, respectively, since previous studies confirmed that L3P and L5P are specific lipopeptides native in S- and C-type MAP strains [35].

Recent assumptions have been proven by PCR IS*1311*-REA *hinf*-1 analysis, in which all three MAP strains (C, S, and bison types) occurred among Sardinian sheep communities. In herd (BTM) group, C-type MAP was the dominant strain (46.67%) rather than S-type (26.67%), bison-type (20%), or an unknown strain (6.6%; this strain had not been reported before). In contrast, S-type was more prevalent (75%) at the individual (MIF) level, as compared with C-type and bison-type, which had the lowest incidences, at 12.5% each. A question that might be raised, here, concerns why C-type MAP, a native strain in cow, should be a common strain among sheep animals at the BTM level. While the S and C types were initially defined as the indigenous strains isolated from, respectively, sheep and bovine, further studies revealed that this host orientation couldn’t inevitably predict the types of MAP strain (S or C) found in milk samples thereof [15]. In fact, genotypic and phenotypic (e.g., pathogenesis and growth pattern) divergences are what could prognosticate S and C types from each other [15,16,17]. Previous group-typing studies depicted that sheep and goat could be infected with both S-type and C-type MAP strains [18,47]. This is due to either an interspecies transmission or the presence of the multiple sources of infection on a given farm [47]. These hypotheses seem logical enough, because BTM is a combination of several individual milk samples that might be infected with either each of or both MAP strains, simultaneously. Furthermore farmers usually keep various species of animals (specifically ruminants) together at the same farm and this could enhance the risk of interspecies transmission among animals in herds.

Although, the distribution of MAP-type strains (S/C) at both the herd and individual levels was accordant with the overall rates of H-MELISA-L3P/L5P positivity, some S- or C-type MAP strains did not follow the predicted patterns in H-MELISA analysis; the majority (44.44%) of S- and C-type MAP strains were detected positive by both H-MELISA L3P and L5P, 22.22% were S type or C type as expected from their positivity patterns in H-MELISA L3P/L5P, and only 11.11% were positive but classified in unexpected categories. Surprisingly, the result of H-MELISA L3P/L5P on BTMs and individual milk samples (MIF) depicted that the majority of bison-type isolates were more reactive against L3P than L5P, at both the herd and individual levels. Previous molecular analysis unveiled that bison-type MAP is derived from a C-type MAP strain that underwent a single nucleotide polymorphism in the IS*1311* sequence [15,19], but complex growth requirements and disease-manifestation patterns distinguished bison-type from C-type MAP [19]. Further investigation on bison strains, isolated from the US and India [15,25], demonstrated that Indian strains have a TG deletion at base pairs 64 and 65 of locus 2 in IS1311 [15,26]. To the best of our knowledge, the tendency of bison-type MAP to reactivity against L3P has never been reported, on which more research is needed to explain this orientation based on the characteristics of lipopeptide in this MAP strain.

At first glance at this recent result, an ambiguity might be found in the stratification of MAP strain types (S or C), based on the result of H-MELISA L3P/L5P. We hypothesized that the antibody reactivity against L3P or L5P might be influenced by either a cross reactivity between the two lipopeptides or a possible co-infection of the studied animals with the two MAP strains. Previous studies straightforwardly showed that MAP strains (S and C types) have various lipopeptides (L3P and L5P) [35]. While the possibility of any cross-reactivity from L5P in mycobacteria close to *Mycobacterium avium complex* (MAC) or other species, such as *M. bovis,* has been rejected [37], the theory of cross-reactivity between L3P and L5P might not be imaginary, since both lipopeptides have common paratopes (e.g., amino acids of D-Phe, N-Methyl-L-Val and L-Ala) between them [35]. However, L3P has been subjected to evolutionary modifications that have specified its structures as distinct from L5P, in which, as a result of mutations (deletions) in S-type MAP, it no longer has two amino acids of L-Ile and L-Phe in its structure [35]. This is under the condition that a study on engineered soluble-in-water L5P revealed that L5P could not differentiate sheep animals that were experimentally infected with subtype I of S-strain MAP from healthy animals. This may represent S-I MAP’s possession of a lipopeptide that is different from L3P (specific lipopeptide in S-III MAP) and L5P (specific lipopeptide in C type MAP) [37].

On the other hand, the possibility of a mixed infection with both MAP (S and C) strains should be examined carefully for the possibility that some samples (specifically BTMs) might contain enough antibodies directed against both L3P and L5P to be recognized positive by both H-MELISA L3P and H-MELISA L5P simultaneously. As the result of RE on amplified IS*1311* fragments confirmed that the three different MAP strains (S, C, and bison types) circulated among animals at the herd and individual levels. Our assumption about the presence of a mixed infection, specifically, in the herd group (BTM), was fortified when a Pearson analysis explained a moderate and low positive correlation between antibody reactivity against L3P and L5P at the herd (BTM; (*r* (45) = 0.5, *p* = 0.00039) and individual levels (MIF; (*r* (79) = 0.37, *p* = 0.00059), respectively. This result indicated that the patterns of antibody reactivity against L3P and L5P are more similar at the BTM level rather than the MIF level.

## 6. Conclusions

MAP-derived lipopeptides, L3P and L5P, could be considered potent capture antigens in serum/milk antibody-detection analyses. Our result suggest that these lipopeptides, in the format of an in-house milk ELISA and as a complementary MAP-screening test, could sensitively predict the infected animals (sheep) at the BTM and individual levels.; it was found that H-MELISA L3P/L5P could enhance the discovery of positive milk samples among the specimens that were potentially negative by other MAP-diagnostic assays. Accordingly, the majority of sheep-milk samples were detected positive by H-MELISA L5P and H-MELISA L3P at the levels of the herd (BTM) and individual sample (MIF) respectively. On the one hand, the overall rates of H-MELISA L3P/L5P confirmed the higher incidence of C-type and S-type classes of MAP in the herd (BTM) and individual (MIF) categories, respectively. Contrary to this overall estimation, some S- or C-type MAP strains did not show the expected reactivity against L3P and L5P, in which either they were positive by both H-MELISA L3P and L5P, or they had reverse reactivity against L3P/L5P. This might express the possibility of either cross-reactivity between L3P and L5P, due to the similarity in the overall structures of their two epitopes, or a within-herd mixed infection of both MAP strains. However, the presence of various MAP strain-types (S/C/Bovine), along with a moderate correlation between antibody reactivity against L3P and L5P that was estimated by Pearson correlation test, urged us to conclude that co-infection with both MAP strains (S and C) happens among the studied animals, at the herd level (BTM). These findings suggest that the L3P and L5P antigens could be useful for improving existing diagnostic tests, especially considering the MAP-strain diversity affecting these animals.

## Figures and Tables

**Table 1:** Distribution of samples of bulk tank milk (BTM) and individual (MIF) milk into two categories, healthy or MAP-positive.

Number/Type of Milk Sample	Number of Negative Control Samples	Number of MAP-Positive Control Samples
47/BTM	17	30
81/Individual (MIF)	45	36

**Table 2:** MAP epitopes used as coating antigens in H-MELISA analyses, and their sequences.

Peptide	Sequence
Lipotripeptide (L3P)	CH_3_-(CH_2_)_18_-CONH-D-Phe-L-Nme-Val-L-Ala-OCH_3_ [36]
Lipopentapeptide (L5P)	CH_3_-(CH_2_)_18_-CONH-D-Phe-L-Nme-Val-L-Ile-L-Phe-L-Ala-OCH_3_ [36]

**Table 3:** Sequences of primer sets used for PCR IS*1311* and qPCR IS*900* analyses of this study.

Primers	Sequences
IS*1311*-F (M56)	5′-GCGTGAGGCTCTGTGGTGAA-3′
IS*1311*-R (M119)	5′-ATGACGACCGCTTGGGAGAC-3′
IS*900*-F (AV1)	5′-ATGTGGTTGCTGTGTTGGATGG-3′
IS*900*-R (AV2)	5′-CCGCCGCAATCAACTCCAG-3′

**Table 4:** Distribution of 23 IS*1311*-positive samples in BTM and MIF categories, based on the type of MAP strain (S/C/bison) and corresponding RE fragments on gel-electrophoresis.

Strain Type	Sample Type		RE ^3^ Fragments on Gel-Electrophoresis Based on Base Pair			
	BTM ^1^ %	MIF ^2^ %	67-bp	218-bp	285-bp	323-bp
S	26.67	75%			+	+
C	46.67	12.5	+	+	+	+
bison	20	12.5	+	+		+

^1^ BTM: bulk tank milk. ^2^ MIF: individual milk samples from a MAP-infected flock. ^3^ RE: restriction Enzyme analysis.

**Table 5:** The distribution of 15 BTMs and 8 individual milk (MIF) samples, based on the types of MAP identified therein by strain-typing analysis and H-MELISA-L3P/L5P positivity/negativity.

Sample Number	Strain Type	H-MELISA L3P (Cutoff = 0.8895) ^1^ (Cutoff = 0.406) ^2^	H-MELISA L5P (Cutoff = 0.59925) ^3^ (Cutoff = 0.513) ^4^
6B	C-type	1.092	0.5795
10B	C-type	0.5545	0.597
12B	Unknown	0.627	0.7505
13B	S-type	0.3865	0.9265
14B	C-type	0.433	0.882
18B	bison	0.7075	0.6585
19B	bison	0.89	0.5605
20B	S-type	0.822	0.842
23B	C-type	0.978	0.885
28B	C-type	0.978	1.311
29B	bison	0.865	0.729
31B	S-type	0.79	0.776
33B	S-type	0.9015	0.6215
79B	C-type	0.755	0.503
82B	C-type	1.006	0.533
7V	S-type	0.4725	0.161
62V	S-type	0.509	0.5155
64V	bison	0.4445	0.312
65V	C-type	0.926	0.6175
72V	S-type	0.418	0.2535
76 V	S-type	0.175	0.3845
83V	S-type	1.062	0.7985
93V	S-type	0.2025	0.2145

^1,2^ Optical densities of 0.8895 and 0.406 were adjusted as the cutoffs corresponding H-MELISA L3P positivity at the BTM (B) and individual (V) levels respectively. ^3,4^ Optical densities of 0.59925 and 0.513 were adjusted as the cutoffs corresponding H-MELISA-L5P positivity at BTM (B) and MIF (V) levels respectively.

## Data Availability

The data presented in this study are available within the article and Supplementary Material section titled Tables S1 and S2.

## Supplementary Materials

The following are available online at https://www.mdpi.com/article/10.3390/vaccines9090997/s1, Table S1: Distribution of 47 bulk tank milk (BTM) samples based on their positivity/negativity status in H-MELISA L3P, H-MELISA L5P, commercial ELISA (CMELISA), and milk qPCR IS900 (MqPCR); Table S2: Distribution of 81 individual milk samples from a MAP-infected flock (MIF) of sheep based on their positivity/negativity status in H-MELISA L3P, H-MELISA L5P, commercial ELISA (CMELISA), milk qPCR IS900 (MqPCR), serum ELISA (SELISA), and fecal PCR (FPCR).

## Abbreviations

MAP	*Mycobacterium avium subsp. paratuberculosis*
JD	Johne’s disease
ELISA	Enzyme-Linked Immunosorbent Assay
S-type	Sheep type
C-type	Cow type
L3P	Lipotripeptide
L5P	Lipopentapeptide
BTM	Bulk Tank Milk
H-MELISA	Homemade Milk ELISA
MIF	MAP-infected Flock
RE	Restriction Enzyme
ROC	Receiver Operating Characteristic
AUC	Area Under the Curve
MqPCR	Milk qPCR
CMELISA	Commercial Milk ELISA
SELISA	Serum ELISA
FPCR	Fecal PCR
OD	Optical Density
SP	Specificity
SN	Sensitivity
