# Supplementary material for: A Comparative Study on the Efficiency of Two Mycobacterium avium subsp. paratuberculosis (MAP)-Derived Lipopeptides of L3P and L5P as Capture Antigens in an In-House Milk ELISA Test
Source: Vaccines (Basel). 2021 Sep 7;9(9):997. doi: 10.3390/vaccines9090997 (PMC8471605; doi:10.3390/vaccines9090997)
Supplement: Supplementary file 1 [file vaccines-09-00997-s001.zip › vaccines-1327424-supplementary.pdf]

Table S1. Distribution of 47 bulk tank milk (BTM) samples based on their positivity/negativity status in H-MELISA L3P, H-MELISA L5P, commercial ELISA (CMELISA), and milk qPCR IS900 (MqPCR).

Number of samples (BTMs)	H-MELISA L3P	H-MELISA L5P	Commercial MELISA (CMELISA)	Milk qPCR IS900 (MqPCR)
Sample 2	-	+	+	-
Sample 3	-	-	+	+
Sample 4	-	+	-	-
Sample 5	-	+	+	-
Sample 6	+	-	+	+
Sample 7	-	+	+	+
Sample 8	-	-	+	+
Sample 9	-	-	-	-
Sample 10	-	+	+	+
Sample 12	-	+	-	+
Sample 13	-	+	+	+
Sample 14	-	+	-	+
Sample 16	-	+	+	+
Sample 17	+	+	-	-
Sample 18	-	+	+	+
Sample 19	+	-	+	+
Sample 20	-	+	-	+
Sample 21	-	-	-	-
Sample 22	-	+	-	+
Sample 23	+	+	-	+
Sample 25	+	+	+	+
Sample 28	+	+	-	+
Sample 29	-	+	-	+
Sample 30	+	+	-	+
Sample 31	-	+	-	+
Sample 32	-	+	-	-
Sample 33	+	+	+	+
Sample 35	-	+	-	-
Sample 47	+	+	+	+
Sample 50	-	+	-	-
Sample 51	-	+	-	-
Sample 52	+	+	-	-
Sample 55	-	+	-	-
Sample 72	-	+	-	-
Sample 73	+	-	+	+
Sample 74	+	-	+	+
Sample 75	-	+	+	+
Sample 79	-	-	+	+
Sample 80	-	-	+	+
Sample 81	-	-	-	-
Sample 82	+	-	-	+
Sample 83	-	-	-	-
Sample 84	-	-	+	+
Sample 85	-	-	-	-
Sample 87	-	-	-	-
Sample 89	-	-	-	-
Sample 91	-	+	-	-

Table S2. Distribution of 81 individual milk samples from a MAP-infected flock (MIF) of sheep based on their positivity/negativity status in H-MELISA L3P, H-MELISA L5P, commercial ELISA (CMELISA), milk qPCR IS900 (MqPCR), serum ELISA (SELISA), and fecal PCR (FPCR).

Number of samples (individual milk samples from a MAP-infected flock (MIF))	H-MELISA L3P	H-MELISA L5P	Commercial MELISA (CMELISA)	Milk qPCR IS900 (MqPCR)	Serum ELISA (SELISA)	Fecal PCR (FPCR)
3	+	-	-	-	-	-
4	+	-	-	-	-	-
5	+	-	+	+	-	+
6	-	-	+	+	+	+
7	+	-	+	+	+	+
8	+	-	+	-	+	+
9	+	-	-	-	-	-
10	+	-	-	-	-	-
11	+	-	-	-	-	-
12	+	-	-	-	-	-
13	-	-	-	-	-	-
15	+	+	-	-	-	-
16	-	-	-	-	-	-
17	+	-	-	-	-	-
18	-	-	-	-	-	-
19	-	-	-	-	-	-
20	-	-	-	-	-	-
22	+	-	+	-	+	-
23	+	-	-	-	-	-
24	+	-	-	-	-	-
25	+	+	+	-	+	-
27	+	-	-	-	-	-
28	-	-	-	-	-	-
29	+	+	-	-	-	-
33	+	-	-	-	-	-
35	+	-	-	-	-	-
36	+	-	+	-	+	+
37	-	-	-	-	-	-
38	+	+	+	-	+	-
39	-	-	-	-	-	-
41	+	-	-	-	-	-
45	-	-	-	-	-	-
49	-	-	+	-	-	-
50	+	+	-	-	+	-
53	+	+	+	-	+	-
55	-	+	+	-	+	-
56	-	-	-	-	+	-
61	+	-	-	-	-	-
62	+	+	+	+	+	-
64	+	-	-	+	+	+
65	+	+	-	+	-	-
66	+	-	-	-	-	-
67	+	-	-	-	-	-
69	-	-	+	+	+	-
70	+	-	+	-	+	-
72	+	-	+	+	+	+
73	+	-	-	-	-	-

75	-	-	-	+	-	-
76	-	-	-	+	-	-
78	+	+	-	-	-	-
79	+	+	-	-	-	-
80	+	-	-	-	-	-
81	+	+	-	-	-	-
82	+	+	+	-	+	+
83	+	+	-	+	-	-
84	+	+	-	-	-	-
86	+	+	-	+	-	-
88	+	-	-	-	+	-
89	+	-	-	-	-	-
90	+	+	-	-	-	-
91	+	-	-	-	-	-
92	+	-	-	-	-	-
93	-	-	+	+	+	-
94	-	+	+	+	+	-
95	+	+	+	-	+	-
96	-	-	+	-	+	-
97	+	+	-	-	+	-
99	+	-	-	+	-	-
100	+	-	-	+	-	-
101	+	-	-	-	-	-
102	+	-	-	-	-	-
103	-	-	-	-	-	-
104	-	-	-	-	-	-
105	+	+	-	-	-	-
111	-	-	-	-	-	-
112	+	+	+	+	+	-
113	+	+	-	+	-	-
114	-	+	+	+	+	-
119	-	+	+	-	+	-
121	+	+	-	-	-	-
128	-	-	-	-	-	-
